# DNA Markers for Detection and Genotyping of *Xanthomonas euroxanthea*

**DOI:** 10.3390/microorganisms10061078

**Published:** 2022-05-24

**Authors:** Kayla Gisela Silva, Leonor Martins, Miguel Teixeira, Joël F. Pothier, Fernando Tavares

**Affiliations:** 1 CIBIO, Centro de Investigação em Biodiversidade e Recursos Genéticos, InBIO Laboratório Associado, Campus de Vairão, Universidade do Porto, 4485-661 Vairão, Portugal; up201706393@fc.up.pt (K.G.S.); miguel.teixeira@ntnu.no (M.T.); 2 Departamento de Biologia, Faculdade de Ciências, Universidade do Porto, 4169-007 Porto, Portugal; 3 BIOPOLIS Program in Genomics, Biodiversity and Land Planning, CIBIO, Campus de Vairão, 4485-661 Vairão, Portugal; 4 Environmental Genomics and Systems Biology Research Group, Institute for Natural Resource Sciences, Zurich University of Applied Sciences (ZHAW), Einsiedlerstrasse 31, 8820 Wädenswil, Switzerland; joel.pothier@zhaw.ch

**Keywords:** *Xanthomonas euroxanthea*, taxa-specific DNA markers, multiplex PCR, comparative genomics, genotyping

## Abstract

*Xanthomonas euroxanthea* is a bacterial species encompassing both pathogenic and non-pathogenic strains and is frequently found colonizing the same host plants as *X. arboricola*. This presents the need to develop a detection and genotyping assay able to track these bacteria in microbial consortia with other xanthomonads. Eight *X. euroxanthea*-specific DNA markers (XEA1-XEA8) were selected by comparative genomics and validated in silico regarding their specificity and consistency using BLASTn, synteny analysis, CG content, codon usage (CAI/eCAI values) and genomic proximity to plasticity determinants. In silico, the selected eight DNA markers were found to be specific and conserved across the genomes of 11 *X. euroxanthea* strains, and in particular, five DNA markers (XEA4, XEA5, XEA6, XEA7 and XEA8) were unfailingly found in these genomes. A multiplex of PCR targeting markers XEA1 (819 bp), XEA8 (648 bp) and XEA5 (295 bp) was shown to successfully detect *X. euroxanthea* down to 1 ng of DNA (per PCR reaction). The topology of trees generated with the concatenated sequences of three markers (XEA5, XEA6 and XEA8) and four housekeeping genes (*gyrB*, *rpoD*, *fyuA* and *acnB*) underlined the equal discriminatory power of these features and thus the suitability of the DNA markers to discriminate *X. euroxanthea* lineages. Overall, this study displays a DNA-marker-based method for the detection and genotyping of *X. euroxanthea* strains, contributing to monitoring for its presence in *X. arboricola*-colonizing habitats. The present study proposes a workflow for the selection of species-specific detection markers. Prospectively, this assay could contribute to unveil alternative host species of *Xanthomonas euroxanthea*; and improve the control of phytopathogenic strains.

## 1. Introduction

Following extensive genotyping and comparative genomics studies performed on walnut-associated bacterial isolates, it has been shown that not all the isolates could be identified as *Xanthomonas arboricola* pv. *juglandis,* the phytopathogen commonly acknowledged as causing walnut bacterial blight (WBB) [1,2,3]. Further studies showed that some walnut-associated *Xanthomonas* isolates were taxonomically distinct from any of the other described *Xanthomonas* species.

These were proposed as members of the new species *Xanthomonas euroxanthea* [4]. Pathogenicity assays indicated that *X. euroxanthea* encompasses non-pathogenic and pathogenic strains that can cause WBB-like symptoms, being in a privileged position to investigate genetic determinants of pathogenesis and its evolution [4,5,6]. 

Interestingly, apart from its occurrence in walnuts (*Juglans regia*), recent evidence was gathered reporting the isolation of *X. euroxanthea* from distinct plant host species, such as *Carya illinoensis* (pecan; strains CPBF 761 and CPBF 766), that together with walnut (*Juglans regia*; strains CPBF 367, CPBF 424^T^, CPBF 426 and CFBP 7653) belong to the Juglandaceae family; *Solanum lycopersicum* (tomato plants; strains BRIP 62409, BRIP 62411, BRIP 62415 and BRIP 62418) a member of the Solanaceae family [7,8,9]; and *Phaseolus vulgaris* (common bean; strain CFBP 7622, previously misclassified as *X. arboricola* [10,11]), a Fabaceae plant species. 

More strikingly is that *X. arboricola* strains were also isolated from all the mentioned plant species, suggesting that both *X. euroxanthea* and *X. arboricola* share the same host plants, including the same plant specimen, which raises questions regarding co-colonization and niche-specific adaptations [7,8,12]. Still, taking into consideration the recent taxonomic refinement of *X. arboricola* species and the unearthing of six new *X. euroxanthea* strains (2949, 2955, 2957, 2974, 3640 and F2) [11], it is foreseeable the identification of additional *X. euroxanthea* isolates from plant species that have passed unnoticed so far.

Altogether, the common ecological niche of these two closely related species, plus their cosmopolitan distribution and co-occurrence in different host plant species resulted in misclassifying some *X. euroxanthea* isolates as *X. arboricola* [8,10,11], which calls for the need to develop methods for the detection and genotyping of this bacterial species. Over the years, different diagnostic and molecular typing tools have been developed for a variety of xanthomonads [13], namely for *X. arboricola* including their most studied pathovars *juglandis* [14,15] and *pruni* [16,17,18,19], aiming to address their diversity within a geographic or epidemiological context. 

Generally, these approaches consist of culture-based detection of the phytopathogen by PCR, followed by multilocus sequence analysis (MLSA) of several housekeeping genes to define haplotypes. In fact, multiplex-PCR and dot-blot hybridization assays showed that three *X. arboricola* pv. *juglandis* specific DNA markers (XAJ1, XAJ6 and XAJ8) were absent from *X. euroxanthea* strains CPBF 367, CPBF 424^T^ and CPBF 426, which formed a distinct MLSA cluster [12,14]. 

A robust method to detect *X. euroxanthea* while undoubtedly distinguishing it from *X. arboricola* has not been described so far. Furthermore, while several genotyping techniques have been used to assess the diversity of phytosanitary regulated *Xanthomonas,* as recently reviewed [13], the data currently available regarding genotyping of *X. euroxanthea* is scarce and limited to MLSA studies performed on *Xanthomonas* isolated from walnut trees [12].

In this study, comparative genomics and in silico validation tools were combined as previously described [20] to select eight *X. euroxanthea*-specific DNA markers located in conserved genomic regions. While XEA1, XEA5 and XEA8 DNA markers were chosen to optimize a multiplex PCR detection method for the reliable detection of the target bacteria, altogether the number of SNPs recorded for DNA markers XEA5, XEA6 and XEA8 within the studied *X. euroxanthea* strains, revealed an allelic variation capable to discriminate *X. euroxanthea* strains as efficiently as the housekeeping genes used in MLSA. Ultimately, this work may unlock the possibility to conciliate bacterial detection and diversity assessment using the same DNA markers, which may be particularly useful to survey *X. euroxanthea* populations in environmental samples.

## 2. Materials and Methods

### 2.1. In Silico Selection and Validation of X. euroxanthea-Specific DNA Markers

A synteny analysis of 11 *X. euroxanthea* strains and other 24 representative xanthomonads, downloaded from the NCBI database (Table 1), was performed using MaGe v3.15.3 [21]. This led to the identification of *X. euroxanthea*-specific coding DNA sequences (CDSs) that are concomitantly present in *X. euroxanthea* genomes and absent from non-*X. euroxanthea* strains.

Then, a BLAST was performed in NCBI (accessed in 22 November 2021 at https://www.ncbi.nlm.nih.gov/) using as query the putative *X. euroxanthea*-specific CDSs and the database nr/nt to further confirm their specificity. Sequences with hits pertaining solely to *X. euroxanthea* were considered putative *X. euroxanthea*-specific genomic regions (Table S1) and were used for primer and specific DNA marker design using Geneious^®^ 9.1.8 [22]. The affinity and complementarity of these primers to target *X. euroxanthea* CDSs was checked using NCBI’s Primer-BLAST tool [22,23]. The obtained eight DNA markers were then evaluated for specificity by a BLASTn analysis in Geneious^®^ 9.1.8 and NCBI (using the database nr/nt).

**Figure 1:**
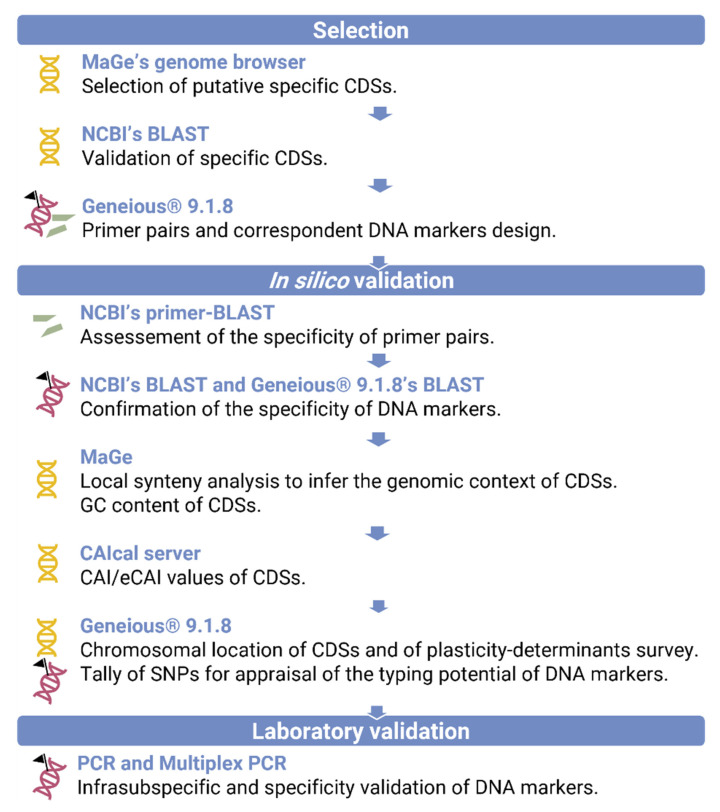
Flowchart for the selection and validation of *X. euroxanthea*-specific DNA markers.

The genomic context of the markers was assessed to ensure its consistency across the diversity of *X. euroxanthea* strains. Particularly, a comparative genomics analysis was performed by local alignment of CDSs in MaGe (Figure S1a–d), to evaluate the syntenic context. Additionally, features, such as the CAI/eCAI values [24]; GC content (deducted from MaGe); chromosomal location; and proximity to determinants of genomic plasticity, namely transposons, integrases, recombinases and phage-related ORFs (Geneious^®^ 9.1.8), were annotated. The number of Single Nucleotide Polymorphisms (SNPs) included in the eight DNA markers and housekeeping genes of the 11 *X. euroxanthea* genomes considered in this study were summed up recurring to Geneious^®^ 9.1.8 and normalized using the formula $\left(\frac{\mathrm{SNPs}\;\mathrm{number}}{\mathrm{total}\;\mathrm{nucleotide}\;\mathrm{sequence}\;\mathrm{length}}\times 100\right)$. The described procedure followed for specific *X. euroxanthea* DNA markers design is systematized in Figure 1.

### 2.2. Bacterial Strains, Culture Conditions and DNA Extraction

The bacterial strains used for the validation of the eight *X. euroxanthea*-specific markers are listed in Table 2 and include seven *X. euroxanthea* strains; and other closely related and niche-sharing strains, namely 10 strains of *X. arboricola*, seven strains representing six pathovars of *X. arboricola* and 11 strains belonging to non-*arboricola Xanthomonas* species. Bacterial strains were cultured as previously described [14] or in peptone-sucrose-agar (PSA) medium (10 g peptone; 10 g sucrose; 1 g glutamic acid; 15 g agar and distilled water up to 1.0 L) at 28 °C. DNA was extracted from pure cultures using the EZNA Bacterial DNA Purification kit (Omega Bio-Tek, Norcross, GA, USA), according to the manufacturer’s instructions and quantified using a DS-11 microvolume spectrophotometer (DeNovix, Wilmington, DE, USA).

### 2.3. Experimental Validation of Putative X. euroxanthea-Specific DNA Markers by Multiplex PCR

A multiplex PCR targeting the most promising DNA markers was optimized to validate a method to rapidly identify *X. euroxanthea* isolates. XEA1, XEA5 and XEA8 were the chosen markers with distinct amplicon lengths of 819, 295 and 648 bp, respectively; and their broad occurrence in the tested *X. euroxanthea* strains, apart from XEA1 (absent in CFBP 7622). A 20 µL PCR reaction mix consisted of 1 × DreamTaq Buffer (ThermoFisher Scientific, Waltham, MA, USA), 0.2 mM of each deoxynucleotide triphosphate (dNTP) (Grisp, Porto, Portugal), 0.2 mM of each forward and reverse primers (Table 3), 1.5 U of DreamTaq DNA Polymerase (ThermoFisher Scientific, Waltham, MA, USA) and 25 ng of DNA template. 

Sterile distilled water was used as the negative control. PCR cycling parameters consisted of a first amplification cycle of 5 min at 95 °C, followed by 35 cycles of 95 °C for 30 s, 61 °C for 15 s and 72 °C for 30 s as well as a final DNA extension at 72 °C for 10 min. The same DNA samples were used as template in PCR reactions using each of the markers individually (XEA1, XEA5 and XEA8) and 1.0 U of DreamTaq DNA polymerase per PCR reaction. 

PCR products were separated by electrophoresis on a 0.8% agarose gel (1 × TAE buffer) and visualized using Xpert Green DNA stain (Grisp, Porto, Portugal) with a Molecular Imager Gel Doc XR+ System (Bio-Rad, Hercules, CA, USA). The obtained PCR products for each marker and each strain were purified using the Illustra GFX GEL Band Purification kit (GE Healthcare, Buckinghamshire, UK), following the reference protocol available and sequenced on both strands (STAB Vida, Caparica, Portugal) to confirm their identity and determine the number of SNPs.

### 2.4. PCR Detection Limit

The detection limit of the multiplex PCR was determined using 10 μL from each of the 10-fold dilutions of *X. euroxanthea* CPBF 424^T^ chromosomal DNA prepared in distilled sterile water, ranging from 100 ng to 1 pg per PCR reaction. Multiplex PCR conditions were kept as described above.

### 2.5. Typing Potential of X. euroxanthea-Specific DNA Markers

Unrooted trees using XEA5, XEA6 and XEA8 markers (transversal to all strains of *X. euroxanthea*) (Table S2) and partial sequence analysis of the housekeeping genes *acnB*, *fyuA*, *gyrB* and *rpoD* (Table S3) of 11 *X. euroxanthea* strains were built to infer the typing potential of these DNA markers. Markers XEA5 (295 bp), XEA6 (237 bp) and XEA8 (648 bp) sequences were concatenated using the Geneious^®^ v. 9.1.7 and used to build a maximum-likelihood tree based on Tamura-Nei model on MEGA X [25], as previously described [14]. 

The nucleotide sequences of housekeeping genes *acnB*, *fyuA*, *gyrB* and *rpoD* were retrieved from the 11 *X. euroxanthea* genomes, aligned and trimmed to 513, 640, 828 and 793 bp, respectively, and subsequently concatenated using the Geneious^®^ v. 9.1.7 to build a maximum-likelihood tree as described for the markers. Since these three markers are *X. euroxanthea* specific, and no homologous marker could be found to define a coherent outgroup, the relatedness of the *X. euroxanthea* strains was inferred by an unrooted tree.

## 3. Results

### 3.1. In Silico Selection of DNA Markers for X. euroxanthea

The selection of *X. euroxanthea*-specific DNA markers was conducted according to the workflow detailed in Figure 1, which consisted on the use of a platform of comparative genomics (Mage) to screen for unique *X. euroxanthea* CDSs, further validated by BLASTn.

A list of CDSs exclusively present in the genomes of 11 *X. euroxanthea* strains and absent from other 12 *Xanthomonas* species, including the closely related *X. arboricola*, was deducted from synteny analysis between the 24 genomes (Table 1) along the full length of *X. euroxanthea* CPBF 424^T^ genome used as reference (Figure S1). The candidate CDSs (from the reference genome of *X. euroxanthea* CPBF 424^T^) were submitted to a BLASTn to confirm their exclusivity in *X. euroxanthea* considering high stringency values of E-value, percentage of identity and query coverage and their suitability as *X. euroxanthea*-specific DNA markers appraised by the design of robust primers. 

**Figure 2:**
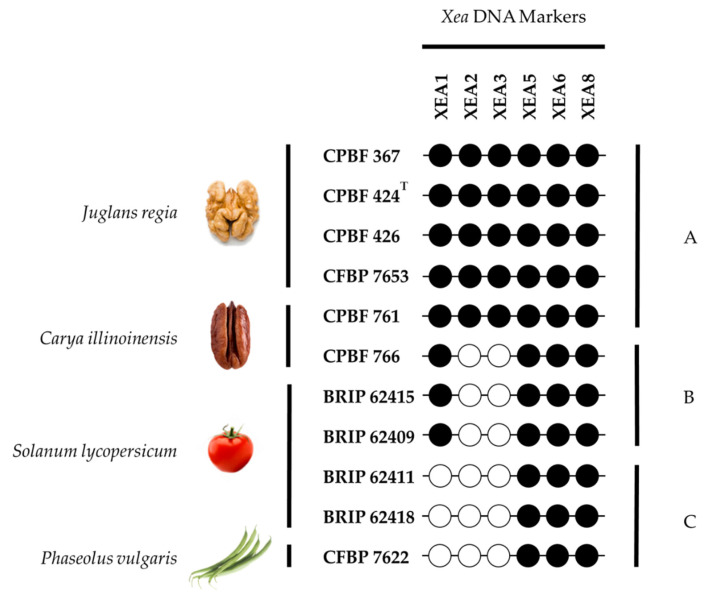
Distribution of six *Xanthomonas* *euroxanthea (Xea)*-specific DNA markers (XEA1, XEA2, XEA3, XEA5, XEA6 and XEA8) in 11 *X. euroxanthea* genomes. The presence/absence of six XEA DNA markers was assessed by BLASTn analysis in Geneious, allowing to disclose three patterns, A to C, that do not translate strain-host affinities.

The data showed that, four CDSs had no significant BLAST hits; three CDSs showed no hits with other *Xanthomonas* sp. and solely one CDS coding for a putative conserved protein of unknown function matched with *Xanthomonas* sp. GW (E-value of 2 × 10^−4^ and query coverage of 19%) (Table 3). These seven CDSs were selected for the design of eight DNA markers, designated XEA1-XEA8; being that two consecutive and partly overlapping CDSs (XE424_v1_a1415 and XE424_v1_a1414) were used to design marker XEA8 (Table 3). Five of these CDSs are predicted to be proteins of unknown function and two to be transcriptional regulators (Table 3). Ultimately, five DNA markers, XEA4-XEA8, are unfailingly present in the genome of the 11 *X. euroxanthea* strains analyzed; XEA1 is present in eight genomes; and XEA2 and XEA3 are present in five strains (Figure 2).

### 3.2. Genomic Analysis Unearths the Stability of XEA DNA Markers

**Figure 3:**
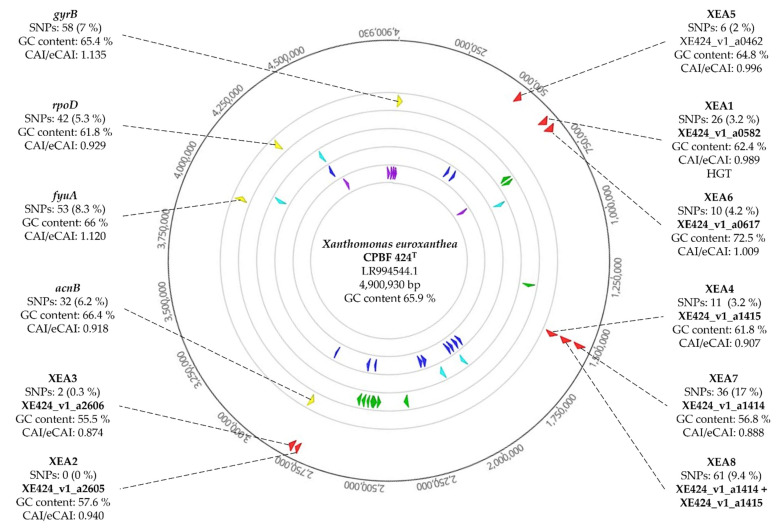
Circular map of *Xanthomonas euroxanthea* strain CPBF 424^T^ chromosome. Outside to inner circles are showing genome coordinates (bp); *X. euroxanthea*-specific DNA markers XEA1-XEA8 (red); housekeeping genes *gyrB*, *rpoD*, *fyuA* and *acnB* (yellow); transposases (green); recombinases (light blue); integrases (dark blue) and phage-related ORFs (purple). For each XEA DNA marker and housekeeping gene the number of SNPs (calculated based on 11 genomes of *X. euroxanthea*), GC content and CAI/eCAI values are shown.

To further assess the uniqueness of these *X. euroxanthea*-specific CDSs used for DNA marker design, several features, including SNPs number, CAI/eCAI values, GC content, chromosomal location and chromosomal proximity to genomic plasticity-determinants were surveyed (Figure 3).

Within the 11 *X. euroxanthea* genomes considered in this study, the number of SNPs for each marker and housekeeping gene was determined and normalized to the full length of the sequence (i.e., DNA marker or housekeeping gene) as a percentual value indicative of the allelic diversity of the sequences (Figure 3).

CAI/eCAI values of CDSs range between 0.874 (for XE424_v1_a2606 corresponding to DNA marker XEA3) to 1.009 (for XE424_v1_a0617 corresponding to DNA marker XEA6); which are values similar to the range attained with housekeeping genes, namely, 0.918 (*acnB*) to 1.135 (*gyrB*) (Figure 3). GC content values for *X. euroxanthea*-specific CDSs varied between 55.5% (XE424_v1_a2606, XEA3) to 72.5% (XE424_v1_a0617, XEA6), which parallels with *X. euroxanthea* CPBF 424^T^ genome GC content value of 65.9% (Figure 3).

Moreover, chromosomal location studies revealed that the XEA markers are scattered throughout the first half of the chromosome and not in the vicinity of genomic plasticity-determinants, including transposases, recombinases, integrases and phage-related ORFs (Figure 3).

**Figure 4:**
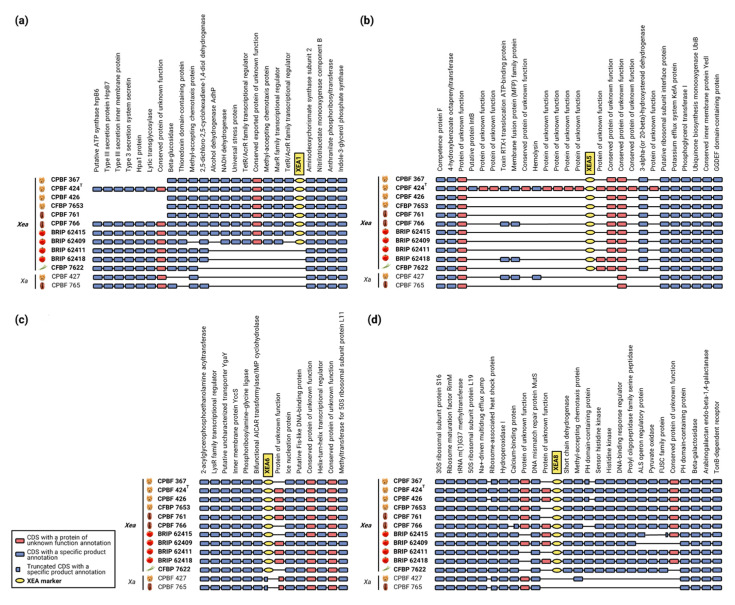
Comparative syntenic maps of four *Xanthomonas euroxanthea*-specific DNA marker-harboring-regions (**a**) XEA1 (designed from a conserved protein of unknown function sequence), (**b**) XEA5 (design from a MarR family transcriptional regulator), (**c**) XEA6 (designed from a conserved protein of unknown function sequence) and (**d**) XEA8 (designed from a protein of unknown function and a TetR/AcrR family transcriptional regulator sequences) DNA markers across 11 *X. euroxanthea* (*Xea*) and two *Xanthomonas arboricola (Xa)* strains.

The synteny analysis performed with MaGe allowed to investigate the genomic context of the most representative DNA markers, i.e., XEA1, XEA5, XEA6 and XEA8, across 11 *X. euroxanthea* genomes and comparatively to *Xanthomonas arboricola* strains CPBF 427 (*X. arboricola* pv. *juglandis*) and CPBF 765 (Figure 4) and other *Xanthomonas* spp. representative strains (Figure S1).

The data showed that XEA1, XEA5, XEA6 and XEA8 are located in highly syntenic regions across all the *X. euroxanthea* genomes studied, underlining the absence of genomic rearrangements (Figure 4). Furthermore, CDSs used for the design of markers XEA5, XEA6 and XEA8 are exclusive of *X. euroxanthea*, being either absent (CDSs encompassing XEA5 and XEA8) or truncated (CDS encompassing XEA6) in *X. arboricola* strains (Figure 4). 

Particularly, the region upstream and including marker XEA1 appears to have suffered erosion in the *X. arboricola* strains analyzed (CPBF 427 and CPBF 765) and in three of the *X. euroxanthea* strains studied (BRIP 62411, BRIP 62418 and CFBP 7622). In parallel, this scenario is observed for the region upstream of XEA5 for all strains, except for CPBF 424^T^; and downstream of XEA8 for the non-*X. euroxanthea* strains.

Within the flanking regions of the XEA markers, the CDSs annotated as unknown proteins are the ones where the presence/absence across strains is inconsistent, suggesting that they have been decaying. While, for XEA6 and XEA8 markers, these events are limited to a single CDS immediately flanking the markers, for XEA5 this paradigm is particularly clear as several CDSs annotated as unknown proteins are present in CPBF 424^T^, have been lost by the other strains.

### 3.3. Multiplex PCR Allows for the Confident Identification of X. euroxanthea Strains

**Figure 5:**
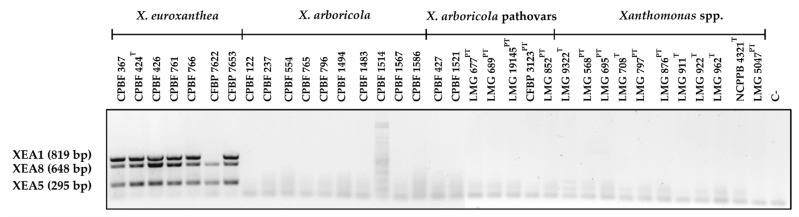
Multiplex PCR using *Xanthomonas euroxanthea*-specific DNA markers XEA1 (819 bp), XEA8 (648 bp) and XEA5 (295 bp) on 7 *X. euroxanthea* strains, 10 *Xanthomonas arboricola* strains, 6 pathovars of *Xanthomonas arboricola* and 9 non-*arboricola Xanthomonas* species. Markers XEA5 and XEA8 were successful in detecting *X. euroxanthea* strains, while XEA1 identified all *X. euroxanthea* strains, except for CFBP 7622. No amplification was observed for any of the other xanthomonads tested, namely *X. arboricola* and other *Xanthomonas* species. C-: negative control.

Reliable identification of *X. euroxanthea* isolates by multiplex PCR was optimized for DNA markers XEA1, XEA5 and XEA8, as these originate amplicons of distinguishable size (819, 295 and 648 bp, respectively) and are located in three independent genomic regions of *X. euroxanthea*. The three markers were successfully amplified in all *X. euroxanthea* strains analyzed, with exception for XEA1 in *X. euroxanthea* CFBP 7622 strain (Figure 5) as expected by in silico studies. In addition, no amplification was observed for any of the other 28 xanthomonads, namely 17 strains of *X. arboricola*, including different pathovars and 9 non-*arboricola Xanthomonas* species.

### 3.4. Detection Limit of Multiplex PCR with XEA DNA Markers

The detection limit of the multiplex PCR targeting the *X. euroxanthea*-specific markers XEA1, XEA5 and XEA8, determined through serial dilution of chromosomal DNA, was 1 ng for PCR reaction (Figure S2). When assessing the PCR detection limit of each marker individually, while for XEA1 and XEA8 the detection limit was identical to the multiplex PCR, i.e., 1 ng/PCR reaction, it was ascertained that the limit of detection lowered to 100 pg/PCR reaction for XEA5.

### 3.5. Typing Potential of Informative XEA DNA Markers

**Figure 6:**
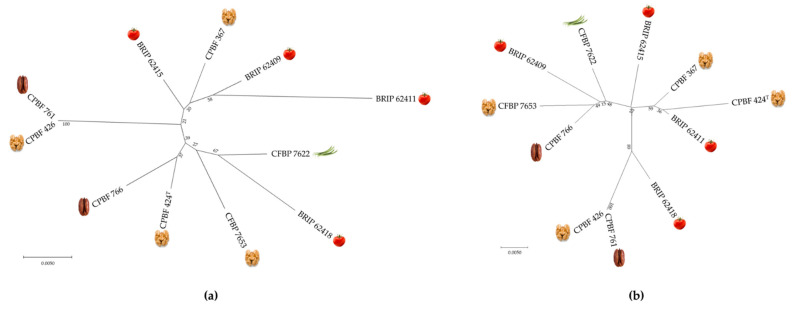
Maximum-likelihood phylogenetic tree based on concatenated sequences of (**a**) DNA markers XEA5, XEA6 and XEA8 (1180 bp); and (**b**) partial housekeeping gene sequences for *acnB*, *fyuA*, *gyrB* and *rpoD* (2774 bp) extracted from 11 *X.* *euroxanthea* genomes. The tree was constructed using the Tamura-Nei model using MEGA X (Kumar et al., 2018 [25]). Supporting values from 1000 bootstrap replicates are indicated near nodes.

The concatenated sequences of XEA5, XEA6 and XEA8 (1180 bp) for each *X. euroxanthea* strain studied were aligned and used to generate a maximum-likelihood tree to investigate the discriminatory potential of these *X. euroxanthea*-specific markers comparatively to four housekeeping genes, corresponding to a concatenated sequence length of 2774 bp, (*acnB*, *fyuA*, *gyrB* and *rpoD* genes) commonly used for MLSA (Figure 6).

Attending that these XEA detection markers are highly specific to *X. euroxanthea*, no homologous sequences have been found in other bacterial taxa, and therefore the allelic diversity determined by the number of SNP (6 SNP/295nt for XEA5, 10 SNP/237nt for XEA6, and 61 SNP/648nt for XEA8; Figure 3) is represented by an unrooted Maximum-likelihood tree. For both trees, each *X. euroxanthea* strain is represented by an independent tree branch, with exceptions for strains CPBF 426 and CPBF 761, which clustered together in a single branch. Furthermore, the topology of both trees does not reflect any clustering according to plant–host of isolation nor by pathogenicity or non-pathogenicity phenotypes (Figure 6).

## 4. Discussion

It is important to investigate the distribution and role played by *X. euroxanthea* and the closely related *X. arboricola* within the plant hosts that they frequently co-colonize [12]. Therefore, it is essential to develop a reliable and efficient method for the accurate detection and identification of *X. euroxanthea* strains and its differentiation from *X. arboricola* [12].

Additionally, early detection is a critical first step towards timely sanitary intervention aimed at eradicating the pathogen and at reducing the inoculum spread to other plants; thus lessening the disease-induced damage in crops from growth to postharvest processing of products and ensuring agricultural sustainability [26]. Likewise, discriminating one bacterial species from the other would be useful in the application of suitable management procedures.

Recommended standard diagnostic protocols from OEPP/EPPO for other plant-diseases caused by *Xanthomonas* spp. (namely, *X. arboricola* pv. *corylina*, *X. axonopodis* pv. *dieffenbachiae*, *X. arboricola* pv. *pruni*, *X. axonopodis* pv. *citri*, *X. fragariae*, *X. oryzae*, *X. axonopodis* pv. *allii*, *X. euvesicatoria*, *X. hortorum pv. gardneri*, *X. perforans* and *X. vesicatoria*) still require several steps-observation of disease symptoms, microscopic examination, pathogen isolation, pathogenicity tests and molecular tests [27,28,29,30,31,32,33,34]. Having in mind that only recently *X. euroxanthea* was proposed as a new species [4], isolated from different plant hosts [7,8,9,10], for which the nature of the bacteria–plant interaction is still unknown, and no distinct symptoms have been described [4], the herein proposed multiplex PCR is the tool available for accurate detection and identification of *X. euroxanthea*.

While multiplex PCRs have been proposed to detect *X. arboricola* pv. *juglandis* [14] or *X. arboricola* pv. *pruni* [35], the present work describes eight *X. euroxanthea-specific* DNA markers (XEA1-XEA8) and proposes a methodology to detect and genotype *X. euroxanthea* strains. In view of that, using a comparative genomics strategy and by assessing a pool of numerous xanthomonads genomes, including several *X. euroxanthea* and *X. arboricola,* it was possible to narrow down *X. euroxanthea*-specific genomic regions to only seven CDSs. Genomes alignment is emerging as a fast and convenient solution to rapidly identify species-specific DNA markers to implement PCR-based detection methods, as recently evidenced for *Xanthomonas campestris* pv. *raphani* [36].

The specificity of these putative *X. euroxathea*-specific DNA markers were further validated by BLASTn analysis using *X. euroxanthea* type strain CPBF 424^T^ as query sequence, followed by an in silico workflow essentially as described in previous studies [20,37], to determine the genomic context of each *X. euroxanthea*-specific loci and particularly to ensure that these putative DNA markers are within conserved and stable genomic regions. 

Specifically, four CDSs showed no significant BLASTn hits outside of *X. euroxanthea*, three CDSs showed some similarity with non *Xanthomonas* species, and only one CDS matched with a *Xanthomonas* spp. but with a poor similarity as shown by an E-value of 2 × 10^−4^ and query coverage of 19%. Based on these data, eight primer pairs corresponding to eight markers (XEA1-XEA8), were designed to nest in these seven CDSs. Marker XEA8 was designed from two consecutive and partly overlapping CDSs. 

Furthermore, the chromosomal distance between each of the *X. euroxanthea* DNA markers (XEA1-XEA8) and genomic plasticity-determinants, such as transposases, recombinases, integrases and phage-related ORFs attests to the low genomic plasticity and high stability of the DNA marker-harboring-regions, as previously suggested [20]. All seven specific CDSs on which XEA markers are founded appear to be well adapted to the codon usage and CG content, thereby, suggesting that these CDSs were not recently acquired by Horizontal Gene Transfer—HGT [20,38]. 

Such findings are consistent with the hypothesis that these CDSs are conserved in the *X. euroxanthea* genomes and likely present across all the *X. euroxanthea* strains regardless of infrasubspecific variability. A BLAST analysis of each DNA-marker (XEA1-XEA8) against the genomes of 11 *X. euroxanthea* showed that five DNA markers (XEA4-XEA8) are unfailingly present in the genome of the 11 *X. euroxanthea* strains analyzed. 

One marker (XEA1) is absent in three out of the 11 *X. euroxanthea* genomes analyzed, and markers XEA2 and XEA3 are present in the four *X. euroxanthea* strains isolated from walnut (CPBF 367, CPBF 424^T^, CPBF 426 and CFBP 7653) and in one strain isolated from pecan (CPBF 766). These results suggest that the CDSs used for the design of markers XEA4-XEA8 are within the core genome of the 11 *X. euroxanthea* studied, whereas the CDSs corresponding to XEA1-XEA3 markers, although *X. euroxanthea*-specific, are part of the accessory genome [39].

An interesting attribute of *X. euroxanthea* is the encompassment of pathogenic (CPBF 424^T^) and non-pathogenic strains (CPBF 367) by the same host [4]. Considering the pathogenicity and non-pathogenicity phenotypes; different plant hosts of isolation; and the occurrence of markers XEA1-XEA8 across the 11 studied *X. euroxanthea*, one may conclude that these *X. euroxanthea*-specific markers are not biased by pathogenicity phenotype or plant host species. 

To determine the allelic variation of the markers and infer their potential to discriminate *X. euroxanthea* strains, the number of SNPs of markers shown to be present in the 11 *X. euroxanthea* strains (i.e., XEA5, XEA6 and XEA8) was determined. The data indicate that XEA8 with a 9.4% of SNPs 61 SNP/648 bp) stands as the most informative *X. euroxanthea*-specific DNA marker for typing purposes, as it surpasses the 2% and 4.2% of SNPs recorded for XEA5 and XEA6 markers, respectively (6 SNP/295 bp for XEA5; and 10 SNP/237 bp for XEA6). 

Although the allelic variation of XEA5 and XEA6 may be inferior to the allelic variation observed for the housekeeping genes commonly used for MLSA (*acnB* (6.2%), *fyuA* (8.3%), *gyrB* (7%) and *rpoD* (5.3%)), when combined with XEA8, it is nevertheless sufficient to separate the 11 *X. euroxanthea* strains as efficiently as the housekeeping genes. These results suggest that these three *X. euroxanthea*-specific DNA markers (XEA5, XEA6 and XEA8) may be used both for the detection and genotyping of *X. euroxanthea*. Since the allelic variation was conducted considering 11 genomes of *X. euroxanthea*, the number of SNPs may be underestimated, i.e., novel *X. euroxanthea* strains may reveal novel single nucleotide substitutions in the sequence of the mentioned markers.

The synteny analyses conducted to investigate the genomic context of the XEA markers across 11 *X. euroxanthea* and two strains of the closely related species *X. arboricola*, revealed that XEA markers and their flanking CDSs are syntenic for *X. euroxanthea*. It is worth mentioning that, within the flanking regions of XEA markers, CDSs annotated as unknown proteins are intermittently absent when compared to CDSs of proteins of known function, suggesting genomic erosion. 

This paradigm is further supported by the genomic context of the XEA5 marker where two CDSs, namely, the toxin RTX-I translocation ATP-binding protein and the membrane fusion protein (MFP) family protein remain present in CPBF 424^T^, CPBF 766, BRIP 62418 and CPBF 427 despite the overall loss of the flanking unknown proteins. The fact that the CDS of the XEA6 marker is present in *X. euroxanthea* strains and truncated in *X. arboricola* strains suggests a common and recent ancestrality of these two species.

Distinctively, markers designed from CDSs annotated as a family transcriptional regulator, namely XEA5 and XEA8, are likely to portray an essential role in cell life and consequently remain conserved in *X. euroxanthea*, which is further supported by their inclusion as part of the core genome.

Overall, the data gathered regarding synteny, CAI/eCAI values and the GC content of *X. euroxanthea*-specific CDSs concerning markers XEA1, XEA5, XEA6 and XEA8, suggest that these markers and their flanking regions are not the result of recent HGT acquisitions events by *X. euroxanthea*, which increases its reliability for *X. euroxanthea* detection and identification, as has been hypothesized for other bacteria [20,37].

A multiplex PCR detection assay targeting XEA1, XEA5 and XEA8 markers was successfully developed for the identification of *X. euroxanthea*. The annealing temperature and extension time were optimized to steer clear of non-specific amplifications of DNA, which was also reportedly been done in previous studies [40]. 

This multiplex PCR was shown to be both specific and efficient given that neither non-specific amplifications (bands of unexpected sizes) in *X. euroxanthea* nor amplification in non-target *Xanthomonas* spp. tested were observed. As predicted by the in silico assessments, the amplicons corresponding to XEA1, XEA5 and XEA8 markers were obtained for all *X. euroxanthea* strains, with the exception of XEA1 for *X. euroxanthea* CFBP 7622. These results emphasize the robustness of the multiplex PCR for the detection and identification of *X. euroxanthea*.

When assessing the PCR detection limit of each marker individually, it was observed that XEA5 lowered the limit of detection value to 10 pg/µL, indicating that the sensitivity of the simplex PCR targeting XEA5 marker is ten-fold higher than the multiplex PCR. The detection limit of 0.1 ng/µL attained for the multiplex PCR is similar to what has been reported in other studies (0.02 and 0.5 ng/µL) [40,41].

To investigate the genotyping potential of the chosen markers, the allelic variation of the three markers present in all studied *X. euroxanthea* strains (XEA5, XEA6 and XEA8) was compared with four housekeeping genes commonly used for MLSA (*acnB*, *fyuA*, *gyrB* and *rpoD*) and represented as unrooted trees. The trees obtained had a similar topology, as each strain was allocated to an independent tree branch, except for strains CPBF 426 and CPBF 761, which clustered together in a single branch, and no SNPs were observed within the concatenated sequences of the XEA markers (1180 bp) nor within the partial housekeeping genes (2774 bp). 

Such data reveals that markers and housekeeping genes are similarly informative in discriminating *X. euroxanthea* strains; and that the studied *X. euroxanthea* strains are not clustered according to plant–host isolation, pathogenicity or non-pathogenicity phenotypes.

By comparing marker-presence profiles to plant host species from which the *X. euroxanthea* strains were isolated, three patterns identified as A, B and C (Figure 2) are observed. Attending that all XEA markers are in syntenic genomic regions that overlap with homologs regions in the closely related *X. arboricola* strains, we may hypothesize that the *X. euroxanthea* strains isolated from two Juglandaceae species (*Juglans regia* and *Carya illinoinensis*) with pattern A, i.e., possessing all XEA markers (XEA1-XEA8), are ancestors of strains included in pattern B (i.e., which lost XEA2 and XEA3) and isolated from *Carya illinoinensis* and Solanaceaceae (*Solanum lycopersicum*) and pattern C (i.e., which lost XEA1, XEA2 and XEA3), isolated from *Solanum lycopersicum* and a Fabaceae (*Phaseolus vulgaris*). 

Thus, rather than an acquisition of XEA2 and XEA3 markers in five of the eleven studied *X. euroxanthea* strains (pattern A), these markers were likely lost in the other *X. euroxanthea* strains, followed by XEA1 marker shown to be absent in 3 out the 11 *X. euroxanthea* strains, leading to the emergence of recent *X. euroxanthea* lineage characterized by marker-profile C (BRIP 62411, BRIP 62418) and *Phaseolus vulgaris* (CFBP 7622). These data suggest that the loss of markers XEA2, XEA3 and XEA1 i.e., patterns A-C, occurred progressively as *X. euroxanthea* lineages extended to a new host, specifically, from walnut, to pecan, to tomato, to the common bean. These results are aligned with studies describing events of genome erosion as a consequence of bacterial adaptation to a new plant–host [42,43].

To summarize, we may infer that the evolutionary trend of XEA marker loss follows progressive host-jump events from walnut, to pecan, to tomato and finally to common bean.

## 5. Conclusions

The present study proposes eight specific, efficient and reliable DNA markers for the detection and identification of *X. euroxanthea* isolates. The allelic variation of some of these markers allows to conciliate the detection and genotyping of *X. euroxanthea* strains, contributing to survey these bacteria in ecological niches colonized by the closely related *X. arboricola*. The multiplex PCR was shown to be highly specific, as solely the target DNA (i.e., *X. euroxanthea*) was amplified; and efficient, as an amplicon was observed with all tested *X. euroxanthea* strains. 

The present study also provided a successful workflow for the selection of molecular markers, which is able to be implemented in the selection of species-specific genomic regions for any other taxa. 

By analyzing a marker’s presence across strains of different colonizing plant host species, we may infer that *X. euroxanthea* colonization of different plant host species occurred at different points in time.

## Figures and Tables

**Table 1:** Bacterial strains used for MaGe synteny analysis to retrieve *Xanthomonas euroxanthea* specific coding sequences (CDSs).

*Xanthomonas* Species and Pathovars	Strains	GenBank, NCBI Accession/WGS Prefix
*X. euroxanthea*	CPBF 367	LR861803.1
*X. euroxanthea*	CPBF 424^T^	LR994544.1
*X. euroxanthea*	CPBF 426	LR861805.1
*X. euroxanthea*	CPBF 761	HG999363.1
*X. euroxanthea*	CPBF 766	HG999364.1
*X. euroxanthea*	CFBP 7622	MIGF01.1
*X. euroxanthea*	CFBP 7653	MIGK01.1
*X. euroxanthea*	BRIP 62409	QEZJ01.1
*X. euroxanthea*	BRIP 624011	QEZI01.1
*X. euroxanthea*	BRIP 62415	QEZH01.1
*X. euroxanthea*	BRIP 62418	QEZG01.1
*X. arboricola*	CPBF 1494	HG999362.1
*X. arboricola*	CPBF 765	HG999365.1
*X. arboricola* pv. *juglandis*	CPBF 427	LR861807.1
*X. campestris* pv. *campestris*	LMG 568^PT^	NC_003902
*X. campestris* pv. *campestris*	8004	NC_007086
*X. campestris* pv. *campestris*	B100	NC_010688
*X. citri* pv. *citri*	306	NC_003919
*X. citri* pv. *bilvae*	NCPPB 3213^PT^	CDHI01
*X. phaseoli* pv. *phaseoli*	CFBP 412	NZ_CP020964.2
*X. citri* subsp. *aurantifolli*	ICPB 10535	ACPY01
*X. vasicola* pv. *musacearum*	BCC282	RRCQ01
*X. vasicola* pv. *musacearum*	NCPPB 4381	ACHT01
*X. oryzae* pv. *oryzae*	PX099^A^	NC_010717.1
*X. oryzae* pv. *oryzae*	MAFF 311018	NC_007705.1
*X. oryzae* pv. *oryzae*	KACC 10331	NC_006834.1
*X. oryzae* pv. *oryzicola*	BLS256	NZ_AAQN
*X. translucens* pv. *translucens*	569	VIWM01.1
*X. translucens* pv. *translucens*	LMG 876^PT^	NZ_CAPJ.1
*X. sacchari*	NCPPB 4393	AGDB01.1
*X. hortorum* pv. *gardneri*	ICMP 7383	CP018731.1
*X. hortorum* pv. *gardneri*	LMG 962^T^	NZ_AEQX.1
*X. vesicatoria*	LMG 911^T^	NZ_AEQV
*X. euvesicatoria* pv. *perforans*	91-118	NZ_AEQW
*X. albilineans*	GPE PC73	NC_013722

**Table 2:** List of bacterial strains used for validation of the *Xanthomonas euroxanthea*-specific DNA markers.

*Xanthomonas* Species and Pathovars	Strains ^1^	Geographic Origin	Year of Isolation
*X. euroxanthea*	CPBF 367	Portugal (Loures)	2016
*X. euroxanthea*	CPBF 424^T^	Portugal (Loures)	2016
*X. euroxanthea*	CPBF 426	Portugal (Loures)	2016
*X. euroxanthea*	CPBF 761	Portugal (Alcobaça)	2016
*X. euroxanthea*	CPBF 766	Portugal (Alcobaça)	2016
*X. euroxanthea*	CFBP 7622	USA	1985
*X. euroxanthea*	CFBP 7653	France	2008
*X. arboricola*	CPBF 122	Portugal (Ponte da Barca)	2015
*X. arboricola*	CPBF 237	Portugal (Ponte de Lima)	2015
*X. arboricola*	CPBF 554	Portugal (Carrazeda de Ansiães)	2016
*X. arboricola*	CPBF 765	Portugal (Alcobaça)	2016
*X. arboricola*	CPBF 796	Portugal (Alcobaça)	2016
*X. arboricola*	CPBF 1494	Portugal (Alcobaça)	2014
*X. arboricola*	CPBF 1483	Portugal (Alcobaça)	2014
*X. arboricola*	CPBF 1514	Portugal (Estremoz)	2014
*X. arboricola*	CPBF 1567	Portugal (Bombarral)	2015
*X. arboricola*	CPBF 1586	Portugal (Loures)	2015
*X. arboricola* pv. *juglandis*	CPBF 427	Portugal (Loures)	2016
*X. arboricola* pv. *juglandis*	CPBF 1521	Portugal (Loures)	2014
*X. arboricola* pv. *celebensis*	LMG 677^PT^	New Zealand	1960
*X. arboricola* pv. *corylina*	LMG 689^PT^	USA	1939
*X. arboricola* pv. *fragariae*	LMG 19145^PT^	Italy	1993
*X. arboricola* pv. *populi*	CFBP 3123^PT^	Netherlands	1979
*X. arboricola* pv. *pruni*	LMG 852^PT^	New Zealand	1953
*X. citri* pv. *citri*	LMG 9322^T^	USA	1989
*X. campestris* pv. *campestris*	LMG 568^PT^	United Kingdom	1957
*X. axonopodis* pv. *dieffenbachiae*	LMG 695^PT^	Brazil	1965
*X. fragariae*	LMG 708^T^	USA	1960
*X. oryzae* pv. *oryzicola*	LMG 797^PT^	Malaysia	1964
*X. translucens* pv. *translucens*	LMG 876^PT^	USA	1933
*X. vesicatoria*	LMG 911^T^	New Zealand	1955
*X. euvesicatoria* pv. *euvesicatoria*	LMG 922	USA	1939
*X. hortorum* pv. *gardneri*	LMG 962^T^	Yugoslavia	1953
*X.euvesicatoria* pv. *perforans*	NCPPB 4321^T^	USA	1933
*X.oryzae* pv. *oryzae*	LMG 5047^PT^	India	1965

^1^ CPBF: Portuguese Collection of Phytopathogenic Bacteria, Instituto Nacional de Investigação Agrária e Veterinária, I.P. Oeiras, Portugal. CFBP: French Collection for Plant-associated Bacteria, Institut National de la Recherche Agronomique, Angers, France. LMG: Belgian Coordinated Collections of Microorganisms/LMG Bacteria Collection, Universiteit Gent—Laboratorium voor Microbiologie, Gent, Belgium. NCPPB: National Collection of Plant Pathogenic Bacteria, Fera Science Ltd., York, UK. Superscript following strain names indicate ^T^ the type strain of a species and ^PT^ the pathotype strain for a pathovar.

**Table 3:** Selected *Xanthomonas euroxanthea*-specific DNA markers (XEA1-XEA8), corresponding primer pair sequences, expected amplicon sizes and best BLASTn hits of amplicons with non-*X. euroxanthea* genomes.

DNA Markers	CDS (MaGe) ^1^	Locus Tag (NCBI)	Gene Annotation (MaGe)	Primers	Sequences (5′→3′)	Length (bp)	Best BLASTn Hit with Non-*X. euroxanthea* (E Value/Query Coverage)
XEA1	XE424_v1_a0582	XTG_000508	Conserved protein of unknown function	XEA1F	CTGCCGAGCGTGAAATCCAG	819	-
				XEA1R	CCTTCAGTTGCACCGAACGC		
XEA2	XE424_v1_a2605	XTG_002379	Conserved protein of unknown function	XEA2F	AGTCCACCAATGCCATCGCC	425	-
				XEA2R	AGTCCACCAATGCCATCGCC		
XEA3	XE424_v1_a2606	XTG_002380	Conserved protein of unknown function	XEA3F	CGGATCGGACAATGACGCTG	612	-
				XEA3R	GCTCTACATCGCCGCTGGAG		
XEA4	XE424_v1_a1415	XTG_001287	TetR/AcrR family transcriptional regulator	XEA4F	GACGCATCCGCCCACGACC	341	*Dickeya zeae* A586-S18-A17 (2 × 10^−50^/95%)
				XEA4R	TAGGCGGCAGACCCCTTCC		
XEA5	XE424_v1_a0462	XTG_000401	MarR family transcriptional regulator	XEA5F	AACGACGCTGACCTGGACC	295	*Sphingomonas* sp. AP4-R1 (5 × 10^−13^/73%)
				XEA5R	CGACACCGCACGACCCCG		
XEA6	XE424_v1_a0617	XTG_000542	Conserved protein of unknown function	XEA6F	GCGGCTGCAGCGTCGTTG	237	*Xanthomonas* sp. GW (2 × 10^−4^/19%)
				XEA6R	TCACCTGATGATCGAAGCCTGG		
XEA7	XE424_v1_a1414	n/a	Protein of unknown function	XEA7F	GGACGCGCCATGATCTGCC	212	-
				XEA7R	GGTGTCCGAGGMTCAGGTGC		
XEA8	^2^	^2^	^2^	XEA8F	ATCGCCTCTGGATGACGGC	648	*Dickeya zeae* A586-S18-A17 (1 × 10^−95^/73%)
				XEA8R	GGTGATGTCGGCAAGCTCG		

n/a: not available; ^1^: no significant hit has been found. ^2^: XEA8 DNA marker was designed within the genomic subsequent and partially overlapping CDSs for markers XEA7 and XEA4.

## Data Availability

Not applicable.

## Supplementary Materials

The following supporting information can be downloaded at: https://www.mdpi.com/article/10.3390/microorganisms10061078/s1, Table S1. MaGe labels of the seven selected *X. euroxanthea*-specific CDSs of 11 *X. euroxanthea* genomes used to design XEA1 to XEA8 markers. Table S2. Genomic coordinates of the eight *X. euroxanthea*-specific DNA markers of 11 *X. euroxanthea* genomes. Table S3. MaGe labels of four housekeeping genes from 11 *X. euroxanthea* genomes used in the construction of an unrooted tree. Figure S1. Synteny map of DNA markers (a) XEA1, (b) XEA5, (c) XEA6 and (d) XEA8 across the genomes of 11 *X. euroxanthea* and 24 other *Xanthomonas* spp. strains. Figure S2. The PCR detection limits assessed using purified DNA from CPBF 424^T^. C-: negative control (sterile distilled water).

## References

[1] Hajri A., Meyer D., Delort F., Guillaumès J., Brin C., Manceau C. (2010). Identification of a genetic lineage within *Xanthomonas arboricola* pv. *juglandis* as the causal agent of vertical oozing canker of Persian (English) walnut in France. Plant Pathol..

[2] Moragrega C., Matias J., Aletà N., Montesinos E., Rovira M. (2011). Apical necrosis and premature drop of Persian (English) walnut fruit caused by *Xanthomonas arboricola* pv. *juglandis*. Plant Dis..

[3] Moragrega C., Özaktan H. (2010). Apical necrosis of persian (english) walnut (*Juglans regia*): An update. J. Plant Pathol..

[4] Martins L., Fernandes C., Blom J., Dia N.C., Pothier J.F., Tavares F. (2020). *Xanthomonas euroxanthea* sp. nov., a new xanthomonad species including pathogenic and non-pathogenic strains of walnut. Int. J. Syst. Evol. Microbiol..

[5] Fernandes C., Blom J., Pothier J.F., Tavares F. (2018). High-Quality Draft Genome Sequence of *Xanthomonas* sp. Strain CPBF 424, a Walnut-Pathogenic Strain with Atypical Features. Microbiol. Resour. Announc..

[6] Teixeira M., Martins L., Fernandes C., Chaves C., Pinto J., Tavares F., Fonseca N.A. (2020). Complete Genome Sequences of Walnut-Associated *Xanthomonas euroxanthea* Strains CPBF 367 and CPBF 426 Obtained by Illumina/Nanopore Hybrid Assembly. Microbiol. Resour. Announc..

[7] Martins L., Teixeira M., Fernandes C., Pothier J.F., Koebnik R., Fonseca N.A., Tavares F. (2022). Genomic Features of Xanthomonas arboricola and X. euroxanthea Sharing the Same Plant Host Species Preferences (Walnut, Pecan and Tomato) Seems Unbiased by the Host Species.

[8] Fernandes C., Martins L., Teixeira M., Blom J., Pothier J.F., Fonseca N.A., Tavares F. (2021). Comparative Genomics of *Xanthomonas euroxanthea* and *Xanthomonas arboricola* pv. *juglandis* Strains Isolated from a Single Walnut Host Tree. Microorganisms.

[9] Roach R., Mann R., Gambley C.G., Shivas R.G., Rodoni B. (2018). Identification of *Xanthomonas* species associated with bacterial leaf spot of tomato, capsicum and chilli crops in eastern Australia. Eur. J. Plant Pathol..

[10] Kałużna M., Fischer-Le Saux M., Pothier J.F., Jacques M., Obradović A., Tavares F., Stefani E. (2021). *Xanthomonas arboricola* pv. *juglandis* and pv. *corylina*: Brothers or distant relatives? Genetic clues, epidemiology, and insights for disease management. Mol. Plant Pathol..

[11] Zarei S., Taghavi S.M., Rahimi T., Mafakheri H., Potnis N., Koebnik R., Saux M.F.-L., Pothier J.F., Palacio-Bielsa A., Cubero J. (2022). Taxonomic Refinement of *Xanthomonas arboricola*. Phytopathology.

[12] Fernandes C., Albuquerque P., Mariz-Ponte N., Cruz L., Tavares F. (2021). Comprehensive diversity assessment of walnut-associated xanthomonads reveal the occurrence of distinct *Xanthomonas arboricola* lineages and of a new species (*Xanthomonas euroxanthea*) within the same tree. Plant Pathol..

[13] Catara V., Cubero J., Pothier J.F., Bosis E., Bragard C., Ðermić E.Ð., Holeva M.C., Jacques M.-A., Petter F., Pruvost O. (2021). Trends in Molecular Diagnosis and Diversity Studies for Phytosanitary Regulated *Xanthomonas*. Microorganisms.

[14] Fernandes C., Albuquerque P., Sousa R., Cruz L., Tavares F. (2017). Multiple DNA Markers for Identification of *Xanthomonas arboricola* pv. *juglandis* Isolates and its Direct Detection in Plant Samples. Plant Dis..

[15] Martins L., Fernandes C., Albuquerque P., Tavares F. (2019). Assessment of *Xanthomonas arboricola* pv. *juglandis* Bacterial Load in Infected Walnut Fruits by Quantitative PCR. Plant Dis..

[16] Pothier J.F., Pagani M.C., Pelludat C., Ritchie D.F., Duffy B. (2011). A duplex-PCR method for species- and pathovar-level identification and detection of the quarantine plant pathogen *Xanthomonas arboricola* pv. *pruni*. J. Microbiol. Methods.

[17] Loreti S., Pucci N., Perez G., Catara V., Scortichini M., Bella P., Ferrante P., Giovanardi D., Stefani E. (2015). Detection and identification of *Xanthomonas arboricola* pv. *pruni* from symptomless plant material: Results of an Italian test performance study. EPPO Bull..

[18] Palacio-Bielsa A., Cubero J., Cambra M.A., Collados R., Berruete I.M., López M.M. (2011). Development of an efficient real-time quantitative PCR protocol for detection of *Xanthomonas arboricola* pv. *pruni* in Prunus species. Appl. Environ. Microbiol..

[19] Palacio-Bielsa A., López-Soriano P., Bühlmann A., van Doorn J., Pham K., Cambra M.A., Berruete I.M., Pothier J.F., Duffy B., Olmos A. (2015). Evaluation of a real-time PCR and a loop-mediated isothermal amplification for detection of *Xanthomonas arboricola* pv. *pruni* in plant tissue samples. J. Microbiol. Methods.

[20] Albuquerque P., Caridade C.M.R., Rodrigues A.S., Marcal A.R.S., Cruz J., Cruz L., Santos C.L., Mendes M.V., Tavares F. (2012). Evolutionary and experimental assessment of novel markers for detection of *Xanthomonas euvesicatoria* in plant samples. PLoS ONE.

[21] Vallenet D., Calteau A., Dubois M., Amours P., Bazin A., Beuvin M., Burlot L., Bussell X., Fouteau S., Gautreau G. (2020). MicroScope: An integrated platform for the annotation and exploration of microbial gene functions through genomic, pangenomic and metabolic comparative analysis. Nucleic Acids Res..

[22] Kearse M., Moir R., Wilson A., Stones-Havas S., Cheung M., Sturrock S., Buxton S., Cooper A., Markowitz S., Duran C. (2012). Geneious Basic: An integrated and extendable desktop software platform for the organization and analysis of sequence data. Bioinformatics.

[23] Stothard P. (2000). The sequence manipulation suite: JavaScript programs for analyzing and formatting protein and DNA sequences. Biotechniques.

[24] Puigbò P., Bravo I.G., Garcia-Vallve S. (2008). CAIcal: A combined set of tools to assess codon usage adaptation. Biol. Direct.

[25] Kumar S., Stecher G., Li M., Knyaz C., Tamura K. (2018). MEGA X: Molecular evolutionary genetics analysis across computing platforms. Mol. Biol. Evol..

[26] Fang Y., Ramasamy R.P. (2015). Current and prospective methods for plant disease detection. Biosensors.

[27] (2021). PM 7/064 (2) *Xanthomonas arboricola* pv. *pruni*. EPPO Bull..

[28] (2004). PM 7/022 (1) *Xanthomonas arboricola* pv. *corylina*. EPPO Bull..

[29] (2009). PM 7/023 (2) *Xanthomonas axonopodis* pv. *dieffenbachiae*. EPPO Bull..

[30] (2005). PM 7/044 (1) *Xanthomonas axonopodis* pv. *citri*. EPPO Bull..

[31] (2006). PM 7/065 (1) *Xanthomonas fragariae*. EPPO Bull..

[32] (2007). PM 7/080 (1) *Xanthomonas oryzae*: Diagnostics. EPPO Bull..

[33] (2016). PM 7/128 (1) *Xanthomonas axonopodis* pv. *allii*. EPPO Bull..

[34] (2013). PM 7/110 (1) *Xanthomonas* spp. (*Xanthomonas euvesicatoria*, *Xanthomonas gardneri*, *Xanthomonas perforans*, *Xanthomonas vesicatoria*) causing bacterial spot of tomato and sweet pepper. EPPO Bull..

[35] Pothier J.F., Vorhölter F.J., Blom J., Goesmann A., Pühler A., Smits T.H.M., Duffy B. (2011). The ubiquitous plasmid pXap41 in the invasive phytopathogen *Xanthomonas arboricola* pv. *pruni*: Complete sequence and comparative genomic analysis. FEMS Microbiol. Lett..

[36] Inoue Y., Fujikawa T., Takikawa Y. (2021). Detection and identification of *Xanthomonas campestris* pv. *campestris* and pv. *raphani* by multiplex polymerase chain reaction using specific primers. Appl. Microbiol. Biotechnol..

[37] Almeida A., Albuquerque P., Araujo R., Ribeiro N., Tavares F. (2013). Detection and discrimination of common bovine mastitis-causing streptococci. Vet. Microbiol..

[38] Puigbò P., Bravo I.G., Garcia-Vallvé S. (2008). E-CAI: A novel server to estimate an expected value of Codon Adaptation Index (eCAI). BMC Bioinform..

[39] Segerman B. (2012). The genetic integrity of bacterial species: The core genome and the accessory genome, two different stories. Front. Cell. Infect. Microbiol..

[40] Fonseca N.P., Felestrino É.B., Caneschi W.L., Sanchez A.B., Cordeiro I.F., Lemes C.G.C., Assis R.A.B., Carvalho F.M.S., Ferro J.A., Varani A.M. (2019). Detection and identification of *Xanthomonas* pathotypes associated with citrus diseases using comparative genomics and multiplex PCR. PeerJ.

[41] Bangratz M., Wonni I., Kini K., Sondo M., Brugidou C., Béna G., Gnacko F., Barro M., Koebnik R., Silué D. (2020). Design of a new multiplex PCR assay for rice pathogenic bacteria detection and its application to infer disease incidence and detect co-infection in rice fields in Burkina Faso. PLoS ONE.

[42] Brown B.P., Wernegreen J.J. (2019). Genomic erosion and extensive horizontal gene transfer in gut-associated Acetobacteraceae. BMC Genom..

[43] Armbruster C.R., Marshall C.W., Garber A.I., Melvin J.A., Zemke A.C., Moore J., Zamora P.F., Li K., Fritz I.L., Manko C.D. (2021). Adaptation and genomic erosion in fragmented *Pseudomonas aeruginosa* populations in the sinuses of people with cystic fibrosis. Cell Rep..

