# Supplementary material for: DNA Markers for Detection and Genotyping of Xanthomonas euroxanthea
Source: Microorganisms. 2022 May 24;10(6):1078. doi: 10.3390/microorganisms10061078 (PMC9227330; doi:10.3390/microorganisms10061078)
Supplement: Supplementary file 1 [file microorganisms-10-01078-s001.zip › microorganisms-1727961-supplementary.pdf]

microorganisms

Article supplementary materials

DNA Markers for Detection and Genotyping of *Xanthomonas euroxanthea*

Kayla G. Silva, Leonor Martins, Miguel Teixeira, Joël F. Pothier and Fernando Tavares

Supplemental tables:

Table S1. MaGe labels of the seven selected *X. euroxanthea*-specific CDSs of 11 *X. euroxanthea* genomes used to design XEA1-XEA8 markers.

Table S2: Genomic coordinates of the eight *X. euroxanthea*-specific DNA markers of 11 *X. euroxanthea* genomes.

Table S3. MaGe labels of four housekeeping genes from 11 *X. euroxanthea* genomes used in the construction of an unrooted tree.

Supplemental figures:

Figure S1. Synteny map of DNA markers (a) XEA1, (b) XEA5, (c) XEA6 and (d) XEA8 across the genomes of 11 *X. euroxanthea* and 24 other *Xanthomonas* spp. strains.

Figure S2. PCR detection limits assessed using purified DNA from CPBF 424^T. C-: negative control (sterile distilled water).

Table S1. MaGe labels of the seven selected *X. euroxanthea*-specific CDSs (for DNA markers design) of 11 *X. euroxanthea* genomes.

***X. euroxanthea* strains**	**MaGe label for CDS used in DNA marker design**						
	**XEA1**	**XEA2**	**XEA3**	**XEA4**	**XEA5**	**XEA6**	**XEA7**
**CPBF 367**	XSP_000481	XSP_002020	XSP_002019	XSP_001326	XSP_000402	XSP_000515	[1]
**CPBF 424**T	XE424_v1_a0582	XE424_v1_a2605	XE424_v1_a2606	XE424_v1_a1415	XE424_v1_a0462	XE424_v1_b0617	XE424_v1_b1414
**CPBF 426**	XSP_000491	XSP_001973	XSP_001972	XSP_001326	XSP_000410	XSP_000525	[1]
**CPBF 761**	XE761_v1_b0564	XE761_v1_b2153	XE761_v1_b2152	XE761_v1_b1437	XE761_v1_b0475	XE761_v1_b0602	[1]
**CPBF 766**	XE766_v1_a0570	-	-	XE766_v1_b1398	XE766_v1_b0459	XE766_v1_b0604	[1]
**CFBP 7622**	-	-	-	MIGF01_270014	MIGF01_300018	MIGF01_80115	[1]
**CFBP 7653**	MIGK01_60160	MIGK01_30158	MIGK01_30157	MIGK01_260023	MIGK01_60023	MIGK01_60196	[1]
**BRIP 62409**	QEZJ01_150401	-	-	QEZJ01_340018	QEZJ01_150518	QEZJ01_150362	QEZJ01_340017
**BRIP 62411**	-	-	-	QEZI01_160016	QEZI01_420165	QEZI01_420020	[1]
**BRIP 62415**	QEZH01_490082	-	-	QEZH01_30015	QEZH01_270036	QEZH01_490118	[1]
**BRIP 62418**	-	-	-	QEZG01_250061	QEZG01_340025	QEZG01_33023	QEZG01_250062

-, CDS absent from this particular genome.

[1] CDS not annotated in MaGe (Geneious® 9.1.8 confirmed the presence of the coding sequences).

Marker XEA8 was designed for the two partly overlapping CDSs used to design markers XEA4 and XEA7.

Table S2. Chromosomal coordinates of the eight *X. euroxanthea*-specific DNA markers of 11 *X. euroxanthea* genomes.

X. euroxanthea strains	Genomic coordinates of DNA markers (bp)							
	XEA1 (819 bp)	XEA2 (425 bp)	XEA3 (612 bp)	XEA4 (341 bp)	XEA5 (295 bp)	XEA6 (237 bp)	XEA7 (212 bp)	XEA8 (648 bp)
**CPBF 367**	570,244-571,062	2,366,790-2,367,214	2,365,643-2,366,254	1,531,285-1,531,625	468,013-467,719	605,286-605,522	1,531,067-1,530,854[3]	1,530,890-1,531,539
**CPBF 424**[T]	611,841-612,659	2,762,517-2,762,093	2,763,664-2,763,053	1,513,728-1,514,068	478,064-477,770	646,704-646,940	1,513,510-1,513,299	1,513,335-1,513,982
**CPBF 426**	579,870-580,688	2,294,375-2,294,799	2,293,228-2,293,839	1,544,553-1,544,893	476,032-475,738	615,051-615,287	1,544,335-1,544,124	1,544,160-1,544,807
**CPBF 761**	579,877-580,695	2,264,312-2,264,736	2,263,165-2,263,776	1,514,531-1,514,871	476,039-475,745	615,058-615,294	1,514,313-1,514,102	1,514,138-1,514,785
**CPBF 766**	605,555-606,373	-	-	1,507,857-1,508,197	471,200-470,906	640,559-640,795	1,507,639-1,507,426	1,507,462-1,508,111
**CFBP 7622**	-	-	-	16,055-16,395	19,751-19,457	121,664-121,900	15,837-15,624	15,660-16,309
**CFBP 7653**	175,010-175,828	178,250-178,674	177,103-177,714	28,344-28,004	20,944-20,650	209,888-210,124	28,562-28,775	28,739-28,090
**BRIP 62409**	444,851-444,042[1]	-	-	15,994-16,334	575,504-575,798	409,763-409,527	15,776-15,562	15,598-16,248
**BRIP 62411**	-	-	-	17,152-17,492	184,923-185,217	19,788-19,552	16,934-16,721	16,757-17,406
**BRIP 62415**	84,958-85,776	-	-	16,009-16,349	52,386-52,680	119,886-120,122	15,791-15,578	15,614-16,263
**BRIP 62418**	-	-	-	67,088-66,748	20,888-20,594	22,667-22,428[2]	67,306-67,517	67,481-66,834

-, DNA marker is absent from this particular genome
[1] XEA1 in the genome of BRIP 62409 has 810 bp

[2] XEA6 in the genome of BRIP 62418 has 240 bp

[3] XEA7 in the genome of CPBF 367 has 214 bp

[4] XEA7 in the genome of CPBF 766 has

Table S3. MaGe labels of four housekeeping genes from 11 *X. euroxanthea* genomes used in the construction of an unrooted tree.

X. euroxanthea strains	Genomic coordinates of housekeeping genes (bp)			
	acnB (513 bp)	*fyuA* (640 bp)	*gyrB* (828 bp)	*rpoD* (793 bp)
**CPBF 367**	2,309,810- 2,310,322	3,978,234-3,978,873	6,525-7,352	4,291,063-4,290,271
**CPBF 424^T**	2,819,809-2,819,297	3,941,493-3,942,132	6,524-7,351	4,277,647-4,276,855
**CPBF 426**	2,238,318-2,238,830	3,807,436-3808,075	6,523-7,350	4,281,395-4,280,603
**CPBF 761**	2,208,262-2,208,774	3,777,389-3778,028	6,523-7,350	4,251,279-4,250,487
**CPBF 766**	2,190,263-2,190,775	3,825,810-3826,449	6,525-7,352	4,200,512-4,199,720
**CFBP 7622**	18,414-18,926	23,387-22,748	4,421-3,594	18,005-18,797
**CFBP 7653**	117,059-117,571	23,271-22,632	74,161-74,988	108,162-108,954
**BRIP 62409**	189,818-190,330	37,662-37,023	87,916-88,743	108,320-109,112
**BRIP 62411**	27,980-28,492	730,566-731,205	104,086-103,259	103,346-104,138
**BRIP 62415**	51,710-51,198	38,083-37,444	125,771-126,598	108,315-109,107
**BRIP 62418**	251,033-250,521	182,168-182,807	197,832-197,005	89,064-88,272

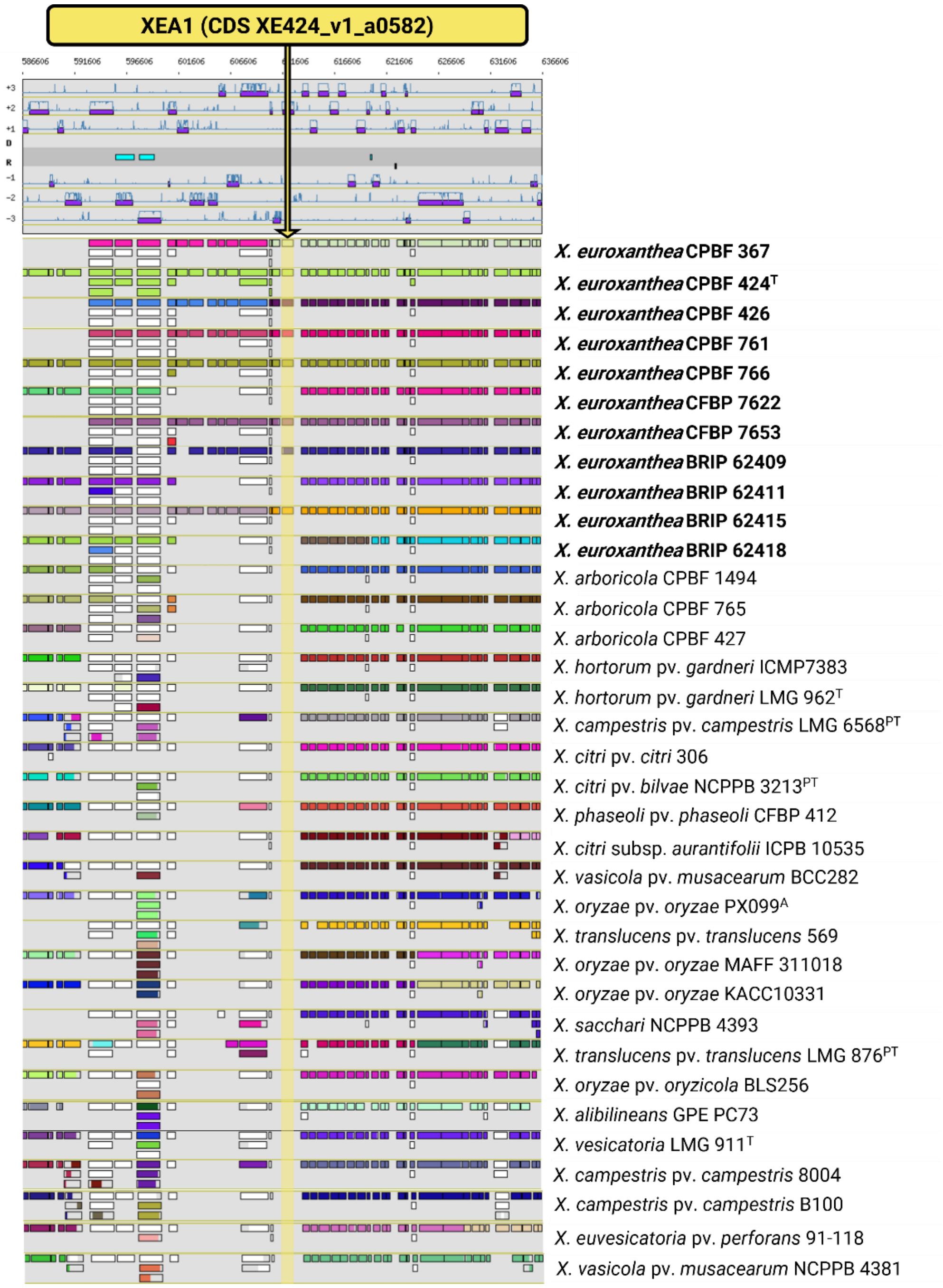

Figure S1(a). Synteny map of XEA1 (highlighted in yellow and designed from a conserved protein of unknown function and its flanking regions) across 11 *X. euroxanthea* strains (CPBF 367, CPBF 424^T, CPBF 426, CPBF 761, CPBF 766, CFBP 7622, CFBP 7653, BRIP 62409, BRIP 62411, BRIP 62415 and BRIP 62418) and 24 other *Xanthomonas* spp. strains.

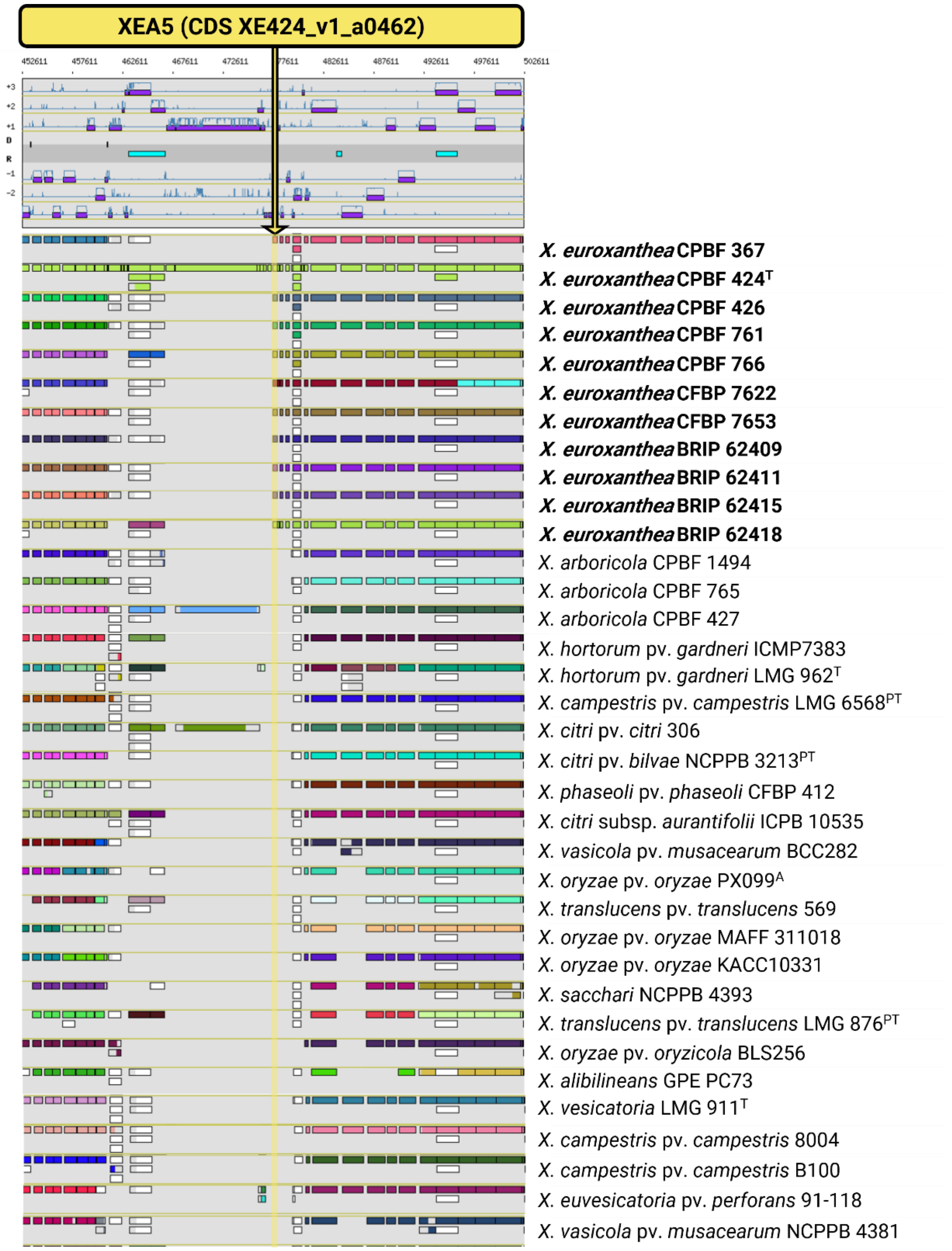

Figure S1(b). Synteny map of XEA5 (highlighted in yellow and designed from a MarR family transcriptional regulator) across 11 *X. euroxanthea* strains (CPBF 367, CPBF 424^T, CPBF 426, CPBF 761, CPBF 766, CFBP 7622, CFBP 7653, BRIP 62409, BRIP 62411, BRIP 62415 and BRIP 62418) and 24 other *Xanthomonas* spp. strains.

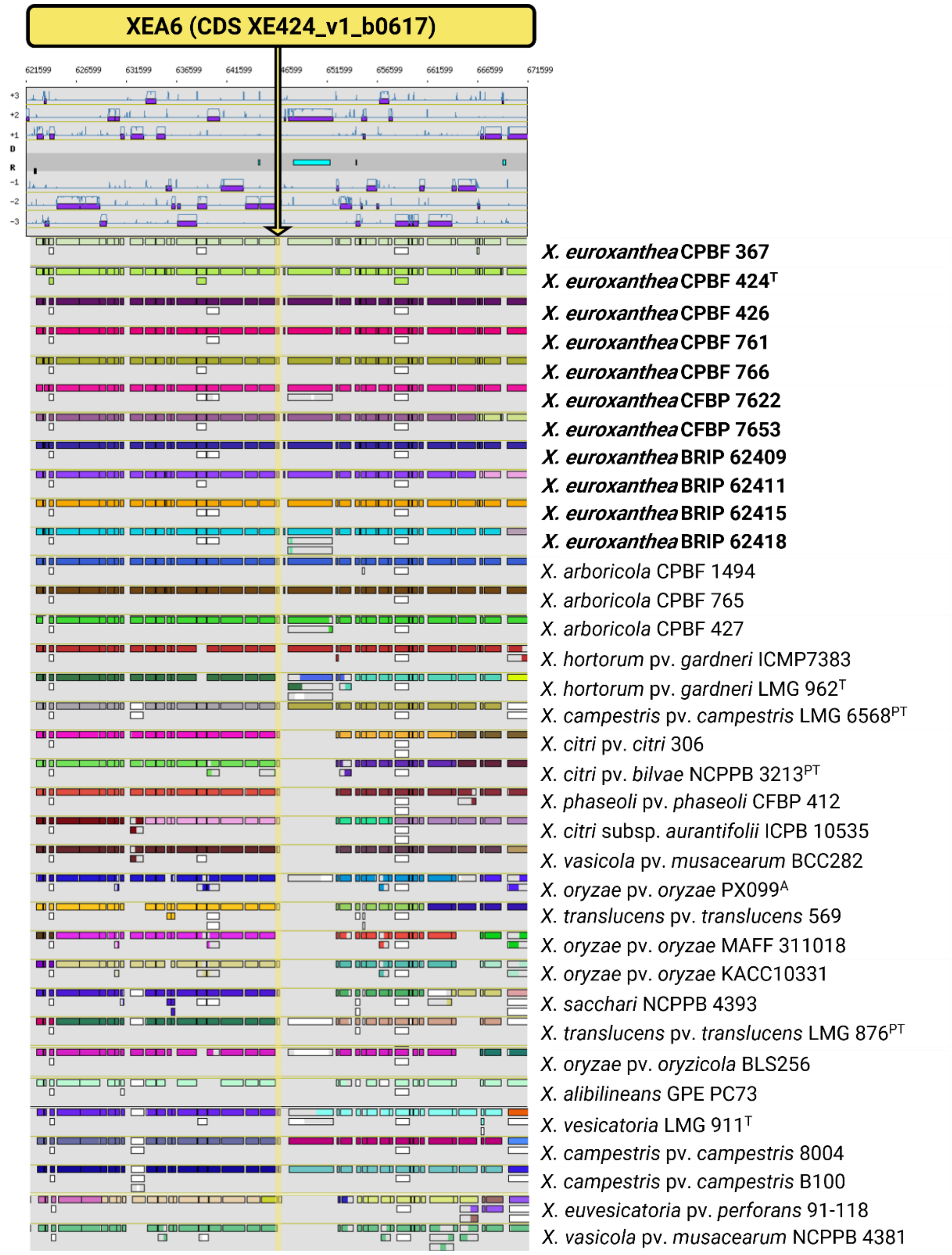

Figure S1(c). Synteny map of XEA6 (highlighted in yellow and designed from a MarR family transcriptional regulator sequence) across 11 *X. euroxanthea* strains (CPBF 367, CPBF 424^T, CPBF 426, CPBF 761, CPBF 766, CFBP 7622, CFBP 7653, BRIP 62409, BRIP 62411, BRIP 62415 and BRIP 62418) and 24 other *Xanthomonas* spp. strains.

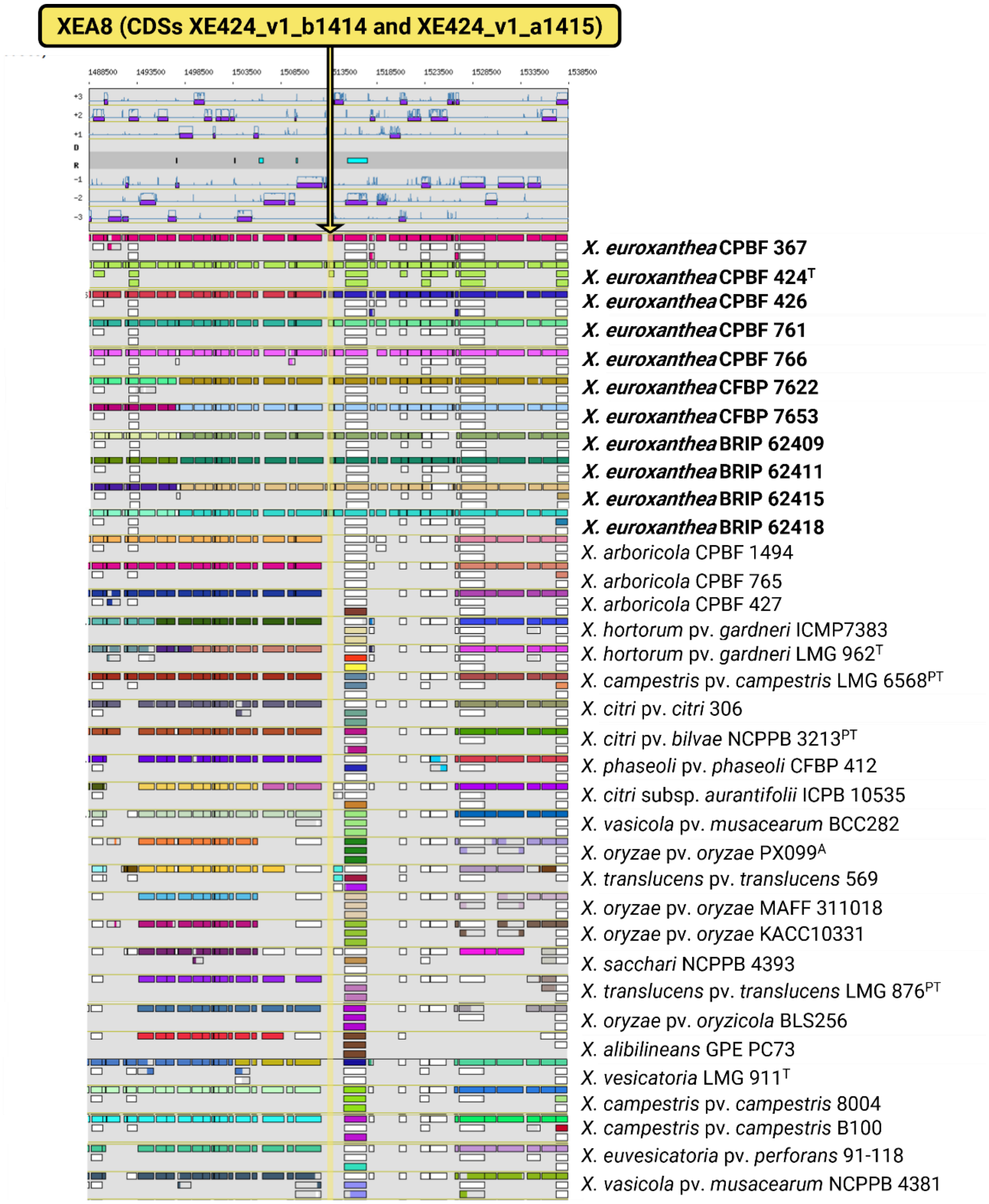

Figure S1(d). Synteny map of XEA8 (highlighted in yellow and designed from a conserved protein of unknown function, and a TetR/AcrR family transcriptional regulator sequences) across 11 *X. euroxanthea* strains (CPBF 367, CPBF 424^{T}, CPBF 426, CPBF 761, CPBF 766, CFBP 7622, CFBP 7653, BRIP 62409, BRIP 62411, BRIP 62415 and BRIP 62418) and 24 other *Xanthomonas* spp. strains.

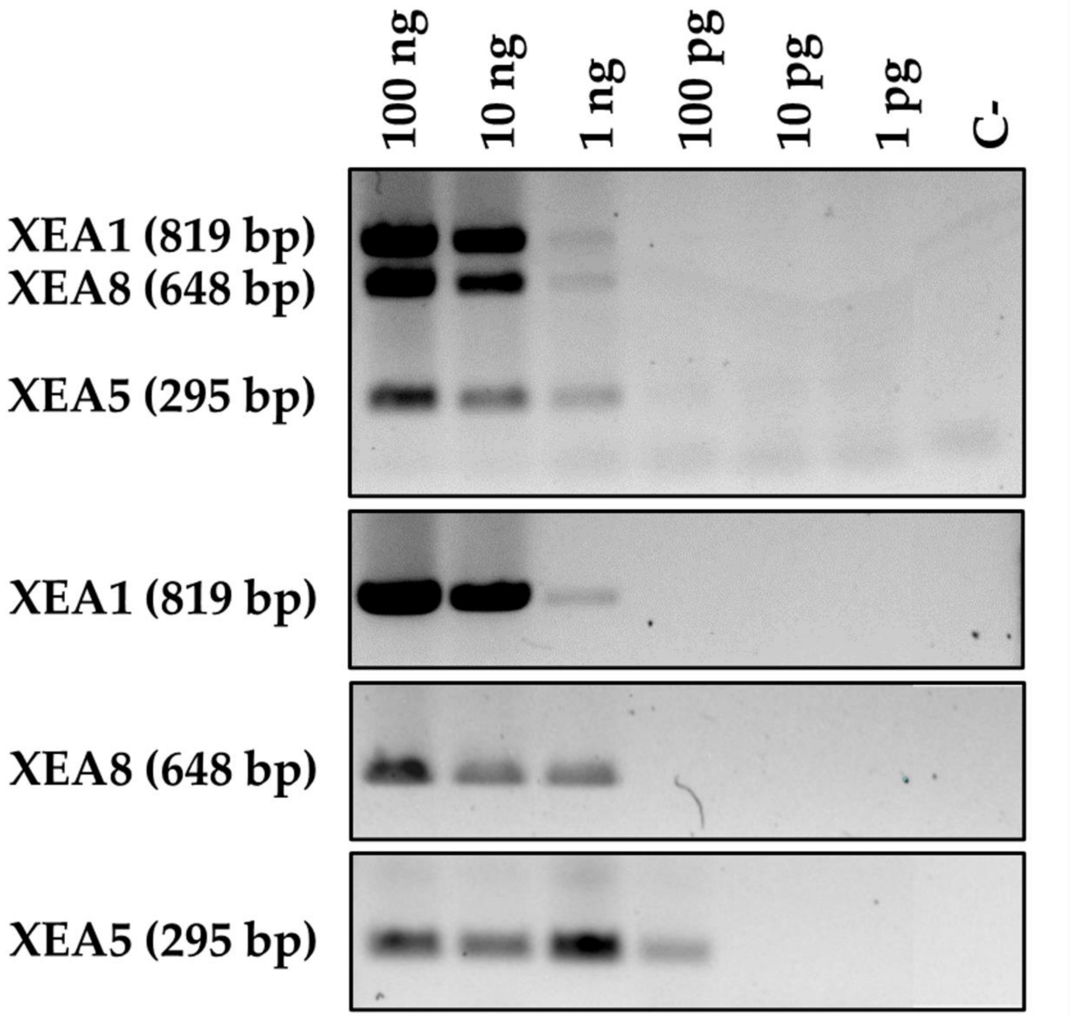

Figure S2. PCR detection limits assessed using purified DNA from CPBF 424^T. C-: negative control (sterile distilled water).
